# Association between interleukin 8–251 T/A and +781 C/T polymorphisms and glioma risk

**DOI:** 10.1186/s13000-015-0378-x

**Published:** 2015-08-07

**Authors:** Hao Liu, Ping Mao, Changhou Xie, Wanfu Xie, Maode Wang, Haitao Jiang

**Affiliations:** Department of Neurosurgery, The First Affiliated Hospital of Xi’an Jiao Tong University, West Yanta Road No.277, Xi’an, 710061 China

## Abstract

**Background:**

Gliomas are aggressive tumors of the central nervous system that rely on production of growth factors for tumor progression. Interleukin 8 (IL-8) is up-regulated in gliomas to promote angiogenesis and proliferation. The aim of this study was to evaluate the association of the IL-8 -251 T/A and +781 C/T polymorphisms and glioma risk.

**Methods:**

We enrolled 300 glioma patients and 300 age- and gender-matched healthy controls. A prospective hospital-based case–control design and logistic regression analysis were utilized. The IL-8 gene polymorphisms were genotyped by polymerase chain reaction-restriction fragment length polymorphism (PCR-RFLP).

**Results:**

Glioma patients had a significantly higher frequency of IL-8 -251 AA genotype [odds ratio (OR) =1.91, 95 % confidence interval (CI) = 1.22, 3.00; *P* = 0.005] and IL-8 -251 A allele (OR =1.36, 95 % CI = 1.08, 1.70; *P* = 0.009) than controls. When stratified by the grade of glioma, patients with WHO IV glioma had a significantly higher frequency of IL-8 -251 AA genotype (OR =1.56, 95 % CI = 1.01, 2.39; *P* = 0.04).

**Conclusions:**

To the best of our knowledge, this is the first report in the literature that the IL-8 -251 AA genotype and A allele were at a higher risk for glioma.

## Background

Gliomas make up about 30 % of all brain and central nervous system tumors and 80 % of all malignant brain tumors [1]. Despite the growing number of preclinical and clinical trials focused on the treatment of malignant gliomas, the prognosis for this disease remains grim [2–4]. Only rare familial syndromes and exposure to high therapeutic doses of ionizing radiation are known causes of glioma [5]. Some other factors are also found to affect glioma risk, such as: high levels of processed meat consumption and obesity during adolescence [6, 7]. Recent research has focused on identifying germline polymorphisms associated with risk of glioma, and using molecular markers to classify glial tumors into more homogenous groups [5, 8, 9].

Interleukin-8 (IL-8), which is best known for its leukocyte chemotactic properties and associated role in inflammatory and infectious diseases [10], is now known to possess tumorigenic and proangiogenic properties as well [11, 12]. IL-8 is encoded by the IL-8 gene located on chromosome 4q13-21, consisting of four exons, three introns, and the proximal promoter region [13]. Several SNPs have been reported in the IL-8 gene and some of them, such as −251 T/A (rs4073) and +781 C/T (rs2227306), can regulate the IL-8 production [14–16]. In human gliomas, IL-8 is expressed and secreted at high levels both *in vitro* and *in vivo*, and recent experiments suggest it is critical to glial tumor neovascularity and progression [12].

We hypothesized that common genetic variants in IL-8 gene influenced the risk of glioma. To test this hypothesis, we performed a prospective hospital-based case–control study to evaluate the association of the IL-8 -251 T/A and +781 C/T polymorphisms and glioma risk.

## Methods

### Study population

In this prospective hospital-based case–control study, we enrolled 300 glioma patients and 300 age- and gender-matched healthy controls between May 2012 and May 2014 in the Department of Neurosurgery, The First Affiliated Hospital of Xi'an Jiao Tong University, China. Tumor type and stage were determined according to the WHO criteria [17]. In addition, similar to the cases the controls were all required to be born in China to native Chinese Han parents. To confirm the diagnosis, two physicians reviewed the hospital records and validated each case. Collected clinical data included sex, age, mean education, smoking, alcohol drinking and family history of cancer. All data points were collected through interviews with the patient or their families/surrogates. All parts of the study were approved by the Institutional Ethical Committee of the First Affiliated Hospital of Xi'an Jiao Tong University, and informed consent according to the Declaration of Helsinki was obtained from all participants or their families/surrogates.

### DNA extraction and genotyping

Genomic DNA was isolated from white blood cells by the commercially available Qiagen kit (QIAGEN Inc., Valencia, CA, USA). IL-8 -251 A/T and +781 C/T polymorphisms were determined by polymerase chain reaction-restriction fragment length polymorphism (PCR-RFLP) as previous described [18–20]. Restriction enzyme and primer sequences of IL-8 promoter SNPs were listed in Table 1. The PCR cycling conditions were 5 min at 94 °C followed by 35 cycles of 1 min at 94 °C, 1 min at 61 °C, and 2 min at 72 °C, with a final step at 72 °C for 20 min. The PCR product was subjected to digestion for 4 h with restriction enzyme at 37 °C. Electrophoresis in a 2.5 % agarose gel followed by ethidium bromide staining and ultraviolet illumination allowed detection of the alleles. For quality control, two independent observers, read all genotypes without knowing about the case or control status. When replicate quality control samples were evaluated, genotypes showed 100 % concordance.

**Table 1:** Restriction enzyme and primer sequences of IL-8 SNPs

Gene and SNP	Enzyme	Forward primer	Reverse primer	Product
IL-8 -251 A/T	*Mfe*I	5′-TCATCCATGATCTTGTTCTAA-3′	5′-GGAAAACGCTGTAGGTCAGA-3′	T/T: 542 bp; A/A: 450 bp, 92 bp
IL-8 + 781 C/T	*Eco*RI	5′-CTCTAACTCTTTATATAGGAATT-3′	5′-GATTGATTTTATCAACAGGCA-3′	T/T: 203 bp; C/C: 184 bp, 19 bp

### Statistical analysis

Data are presented as means ± standard deviation (SD) or as percentages for categorical variables. Differences between continuous variables were assessed by Student’s *t* test, while those between categorical variables were evaluated using Pearson *x*^2^ test. The existence of differences in genotypic frequencies between groups were assessed by means of Pearson *x*^2^ test and calculating the odds ratio (OR) with the 95 % confidence intervals (CI). Statistical significance was taken at nominal *P*-value < 0.05 for all comparisons. SAS version 9.1 (SAS Institute, Cary, NC) was used for all statistical tests.

## Results

### Characteristics of participants

Characteristics of glioma cases and healthy controls were presented in Table 2. No significant differences were found between the glioma cases and healthy controls in sex, age, mean education, smoking, alcohol drinking and family history of cancer (Table 2). Among 300 glioma cases, 223 cases had astrocytomas, 43 cases had ependymomas, 19 cases had oligodendrogliomas, and 15 cases had mixed gliomas (Table 2). In these cases, 17 were WHO grade I gliomas, 106 were WHO grade II gliomas, 76 were WHO grade III gliomas, and 101 were WHO grade IV gliomas (Table 2).

**Table 2:** Characteristics of glioma cases and healthy controls

	Glioma	Controls	*P*
Number of subjects	300	300	
Sex (Male/Female)	165/135	163/137	0.87
Age (years), (mean ± SD)	43.6 ± 9.1	44.1 ± 9.3	0.51
Mean education (years)	10.3 ± 2.3	10.5 ± 2.2	0.28
Smoking (Ever/Never)	124/176	118/182	0.62
Alcohol drinking (Ever/Never)	106/194	102/198	0.73
Family history of cancer (YES/NO)	29/271	27/273	0.78
Histology (%)			
Astrocytomas	223 (74.3)		
Ependymomas	43 (14.3)		
Oligodendrogliomas	19 (6.3)		
Mixed gliomas	15 (5.0)		
WHO Grade (%)			
I	17 (5.7)		
II	106 (35.3)		
III	76 (25.3)		
IV	101 (33.7)		

### IL-8 -251 T/A polymorphisms and glioma risk

Glioma patients had a significantly higher frequency of IL-8 -251 AA genotype (OR =1.91, 95 % CI = 1.22, 3.00; *P* = 0.005) and IL-8 -251 A allele (OR =1.36, 95 % CI = 1.08, 1.70; *P* = 0.009) than controls (Table 3). When stratified by the grade of glioma, patients with WHO IV glioma had a significantly higher frequency of IL-8 -251 AA genotype (OR =1.56, 95 % CI = 1.01, 2.39; *P* = 0.04) (Table 4). When stratified by the histology of glioma, there was no significant difference in the distribution of each genotype (Table 4).

**Table 3:** Genotype and allele frequencies of IL-8 gene polymorphisms among glioma cases and healthy controls

Genotypes	Glioma (%)	Controls (%)	OR (95 %CI)	*P*
−251 TT	96 (32.0)	105 (35.0)	1.00 (Reference)	
−251 TA	120 (40.0)	147 (49.0)	0.89 (0.62,1.29)	0.55
−251 AA	84 (28.0)	48 (16.0)	1.91 (1.22,3.00)	0.005
−251 T allele frequency	312 (52.0)	357 (59.5)	1.00 (Reference)	
−251 A allele frequency	288 (48.0)	243 (40.5)	1.36 (1.08,1.70)	0.009
+781 CC	166 (55.3)	161 (53.7)	1.00 (Reference)	
+781 CT	97 (32.3)	108 (36.0)	0.87 (0.61,1.24)	0.44
+781 TT	37 (12.3)	31 (10.3)	1.16 (0.69,1.96)	0.58
+781 C allele frequency	429 (71.5)	430 (71.7)	1.00 (Reference)	
+781 T allele frequency	171 (28.5)	170 (28.3)	1.01 (0.79,1.30)	0.95

**Table 4:** Stratification analysis of IL-8 -251 T/A polymorphism in glioma

	Cases	TT			TA			AA		
		n (%)	OR (95 %CI)	*P*	n (%)	OR (95 %CI)	*P*	n (%)	OR (95 %CI)	*P*
Histology	300	96 (32.0)	1 (Reference)		120 (40.0)	1 (Reference)		84 (28.0)	1 (Reference)	
Astrocytomas	223	73 (33.6)	1.02 (0.72,1.45)	0.90	88 (39.5)	0.99 (0.71,1.37)	0.94	62 (27.8)	0.99 (0.69,1.44)	0.97
Ependymomas	43	12 (27.9)	0.87 (0.44,1.72)	0.69	18 (41.9)	1.05 (0.58,1.89)	0.88	13 (30.2)	1.08 (0.56,2.10)	0.82
Oligodendrogliomas	19	6 (31.6)	0.99 (0.38,2.54)	0.98	8 (42.1)	1.05 (0.45,2.47)	0.91	5 (26.3)	0.94 (0.34,2.59)	0.91
Mixed gliomas	15	5 (33.3)	1.04 (0.37,2.94)	0.94	6 (40.0)	1.00 (0.38,2.64)	1.00	4 (26.7)	0.95 (0.31,2.95)	0.93
WHO Grade	300	96 (32.0)	1 (Reference)		120 (40.0)	1(Reference)		84 (28.0)	1 (Reference)	
I	17	6 (35.3)	1.10 (0.42,2.88)	0.84	7 (41.2)	1.03 (0.42,2.55)	0.95	4 (23.5)	0.84 (0.28,2.57)	0.76
II	106	34 (32.1)	1.00 (0.64,1.57)	0.99	51 (48.1)	1.20 (0.81,1.79)	0.36	21 (19.8)	0.71 (0.42,1.20)	0.20
III	76	28 (36.8)	1.15 (0.71,1.88)	0.57	33 (43.4)	1.09 (0.69,1.72)	0.73	15 (19.7)	0.71 (0.39,1.29)	0.26
IV	101	28 (27.7)	0.87 (0.54,1.40)	0.56	29 (28.7)	0.72 (0.45,1.14)	0.16	44 (43.6)	1.56 (1.01,2.39)	0.04

### IL-8 + 781 C/T polymorphisms and glioma risk

No association was found between IL-8 + 781 C/T polymorphisms and glioma risk (Table 3).

## Discussion

Recent research has focused on identifying germline polymorphisms associated with risk of glioma. A study by Zong *et al.* suggested that the Cyclin D1 gene G870A polymorphism was a risk factor for the development of glioma [21]. There was a potential decreased susceptibility to glioma in association with the XRCC1 Arg399Gln polymorphism, especially in Asians [22]. Regulator of telomere elongation helicase 1 (RTEL1) rs6010620 polymorphism was likely to be associated with increased glioma risk [23]. A study by Yuan *et al.* suggested that common variants in ERCC1 may contribute to susceptibility to glioma, especially in Asians [24]. The AA genotype of ERCC1 C8092A might be associated with a higher risk of adult glioma than the CA and CC genotypes and that the risk allele of ERCC2 K751Q confered a significant susceptibility to adult glioma, especially in Asian populations [25]. Strong evidence for the association between XRCC3 C18607T polymorphism and glioma risk was found in a meta-analysis [26]. There was a significant association between EGF +61A > G polymorphism and glioma risk among Asians [27]. The polymorphism of interleukin-4 receptor alpha (IL-4Ralpha) rs1801275 played a protective role in the glioma pathogenesis, particularly among Asians [28].

The IL-8 gene polymorphisms were also associated with many other cancers. A recent prospective case–control study found that IL-8 polymorphism was associated with ovarian cancer [29]. The IL-8 -251 AA genotype was at a higher risk for oral cancer in the Caucasian population [30, 31]. The IL-8-251 TA or AA genotype conferred risk of cardia gastric cancer in a population in Southwestern China [32]. The IL-8 -251 AA genotype was associated with the overall risk of developing gastric cancer and may seem to be more susceptible to overall gastric cancer in Asian populations [33]. The IL-8 -251 T/A polymorphism was associated with lung cancer susceptibility in Asians and the −251 A allele may increase risk of lung cancer in Asians [34]. A HuGE review and meta-analysis based on 42 case–control studies found that -251A allele was susceptible in the development of low-penetrance cancers [35]. Two case–control studies found that IL-8 -251 T/A polymorphism was significantly associated with colorectal cancer susceptibility risk [36, 37]. The IL-8-251 T/A polymorphism was associated with breast cancer risk [38]. A study found that IL-8 -251 T/A polymorphism was associated with bladder cancer susceptibility and outcome after bacillus Calmette-Guerin immunotherapy in a northern Indian cohort [39].

The mechanisms of the IL-8 -251 AA genotype and A allele as risk factors of glioma are still unclear. In human gliomas, IL-8 is expressed and secreted at high levels both *in vitro* and *in vivo*, and recent experiments suggest it is critical to glial tumor neovascularity and progression [12]. There is strong evidence that IL-8 secretion is associated with glioma formation and malignant progression [12]. The IL-8 -251A allele in a homozygous state has been associated with increased expression of the IL-8 gene [40, 41]. It is entirely plausible that the the IL-8 -251 AA genotype and A allele will affect the risk of gliomas.

There is limitation that needs to be acknowledged and addressed regarding the present study. First of all, these results should be interpreted with caution because the population was only from China, which reduces the possibility of confounding from ethnicity, so it does not permit extrapolation of the results to other ethnic groups. Second, this study is limited by its size and lack of replication. Additional large scale studies are needed to confirm this finding. Third, since controls were recruited from those who came to hospitals for routine health examination, there was a certain risk of selection bias. Finally, rare familial syndromes and exposure to high therapeutic doses of ionizing radiation are known causes of glioma, which were not explored in the present study.

## Conclusion

To the best of our knowledge, this is the first report in the literature that the IL-8 -251 AA genotype and A allele were at a higher risk for glioma. Additional large scale studies are needed to confirm this finding. Genetic risk profiling has the potential to identify individuals who have not yet had glioma develop but are at high risk. Analysis of polymorphic variants will potentially provide a tool to assess the risk of glioma decades before diagnosis. This process provides ample opportunity to implement behavioral and environmental changes.

### Ethical approval

All procedures performed in studies involving human participants were in accordance with the ethical standards of the institutional and/or national research committee and with the 1964 Helsinki declaration and its later amendments or comparable ethical standards.

### Informed consent

Informed consent was obtained from all individual participants included in the study.
